# Supplementary material for: The decentralization effects of entrepreneurial characteristics on corporate social responsibility
Source: PLoS One. 2022 Nov 28;17(11):e0278030. doi: 10.1371/journal.pone.0278030 (PMC9704557; doi:10.1371/journal.pone.0278030)
Supplement: Attachment — Submitted filename: Response to Reviewers.pdf [file pone.0278030.s001.pdf]

Response to Reviewers

Dear Editors and Reviewers:

Thank you for your letter and for the reviewers' comments concerning our manuscript entitled "The decentralization effects of entrepreneurial characteristics on corporate social responsibility" (PONE-D-22-16972). Those comments are all valuable and very helpful for revising and improving our paper, as well as the important guiding significance to our research. We have studied the comments carefully and have made a correction which we hope meets with approval. Revised portions are marked in red on the paper. The main corrections in the paper and the responses to the reviewer's comments are as flowing:

1. The abstract needs an academic hook, that awakes the interest of the readers.

Response: We have rewritten this part of the abstract according to the Reviewer's comment. The main contributions are as follows:

"The question of whether appropriate decentralization can solve the misplacement of corporate social responsibility (*CSR*) caused by entrepreneurial characteristics (*EC*) is an interesting ethical puzzle. Because corporate behavior depends on the decision-making choices of executives, and their personality characteristics affect the choice tendency. It may be a good way for most businesses to solve the negative problems of *CSR*. "

2. The motivation of the study needs to be clearer.

Response: Based on the negative events of corporate social responsibility (*CSR*), this paper studies the influence of corporate executives' characteristics on the distribution of their decision-making power.

Because the enterprise behavior originates from the executive decision, the executive characteristic is the key factor that affects the decision-making to cause the behavior, which produces the performance result. At the same time, the executive decision-making power decentralization, maybe makes managers not be arbitrary, and reduce negative events.

3. Providing convincing reasons for the controls such as CSR and other variables in the empirical model.

Response: The study mainly involves some controls variables：

"As a result of the above theoretical research and influencing factors of *EC* and *CPDI*, the control variables are taken as the following factors including *Enterprise's Total Assets* (that are owned or controlled for economic benefits), *Employee Size* (refers to the total number of enterprise jobs, including operators, managers, and employees), *Current Assets* (that can be liquidated within a year or a business cycle), *Capital Reserve* (refers to the provident fund formed by the acceptance of donations, the premium of equity and the appreciation of property), *Operating Cost* (refers to the cost of goods or services), and *Asset-liability Ratio* (reflecting the proportion of assets provided by the enterprise creditors). The data of these variables are obtained from the financial statements of listed companies to be taken as logarithms."

4. Consider controlling some corporate governance variables such as the size of the board of directors, auditor quality, board independence, CEO characteristics, etc… (where the data is available)

Response: The variable of "the size of the board of directors", has been calculated for the *CPDI* value, which divides the decision-making power of the company balance between the board and the manager in three steps.

"In the first step, all executives are divided into three groups as director, supervisor, and management team to calculate TPF; Then, using 1, ..., n-1 to represent the seat number in an institution as the leader percentage of each group to calculate PPF; Finally, CPDI is equal to TPF×PPF. Based on previous research, the smaller entropy value represents the more dispersed power of decentralization and authorization for executors."

5. Consider controlling for firm/industry fixed effects. The inclusion of time fixed effect is also encouraged.

Response: In the table of the paper report, *Controls* contains three types of control factors:

(i) One is the control variable, which is respectively *Enterprise's Total Assets*, *Employee Size*, *Current Assets*, *Capital Reserve*, *Operating Cost*, and *Asset-liability Ratio*;

(ii) The second one is the firm fixed effect, which is to control the firm variable;

(ii) The third one is the year fixed effect, that is, control of the time variable.

A sentence has been added at the bottom of Table 2 (The label in the paper): *Controls* include control variables, year fixed effect, and firm fixed effect.

At the same time, the complete regression results are shown in the table below, and we have omitted the control variables section of the paper.

Table 2 The effects of Entrepreneurial Characteristics on Corporate Social Responsibility

**Variables**		**Intrinsic Nature (A)**			**Experience Characteristics (B)**			**Incentive Feature (C)**			**(D)**		
		**(1)*CSR***	**(2)*CPDI***	**(3)*CSR***	**(4)*CSR***	**(5)*CPDI***	**(6)*CSR***	**(7)*CSR***	**(8)*CPDI***	**(9)*CSR***	**(10)*CSR***	**(11)*CSR***	**(12)*CPDI***
Dependent Variable	*Gender*	$0.058^{\#}$ (1.91)	0.017^{***} (6.56)	0.081^{***} (2.72)							0.080^{***} (2.71)		
	Degree (16)				0.125^{*} (2.06)	-0.008^{***} (-3.13)	$0.116^{\#}$ (1.84)				$0.102^{\#}$ (1.74)		
	Degree (19)				0.157^{***} (2.60)	-0.009^{***} (-3.60)	0.147^{*} (2.34)				0.135^{*} (2.31)		
	Salary							0.056^{***} (8.01)	-0.002^{***} (-3.14)	0.054^{***} (7.79)			
Mediator	*CPDI*			-1.345^{***} (-5.74)			-1.233^{***} (-5.33)			-1.008^{***} (-4.36)	-1.072^{***} (-4.54)		

Controls	*Total Assets*	0.075***	-0.002#	0.073***	0.075***	-0.001	0.073***	0.072***	-0.001	0.070***	0.067***	0.077***	-0.002
		(6.29)	(-1.75)	(6.11)	(6.28)	(-1.30)	(6.15)	(6.01)	(-1.25)	(5.91)	(5.66)	(6.38)	(-1.37)
	Employee Size	0.037***	-0.006***	0.029***	0.039***	-0.006***	0.031***	0.033***	-0.006***	0.027***	0.027***	0.038***	-0.006***
		(5.59)	(-8.76)	(4.26)	(5.88)	(-8.63)	(4.67)	(4.98)	(-8.23)	(4.02)	(4.04)	(5.67)	(-8.60)
	Operating Cost	0.051***	0.005***	0.058***	0.049***	0.005***	0.054***	0.045***	0.005***	0.050***	0.051***	0.050***	0.005***
		(6.38)	(6.25)	(7.14)	(6.07)	(5.83)	(6.74)	(5.58)	(6.08)	(6.11)	(6.13)	(6.24)	(5.79)
	Asset-liability Ratio	-0.009	-0.004***	-0.015	-0.009	-0.004***	-0.015	-0.003	-0.005***	-0.007	-0.009	-0.008	-0.004***
		(-0.84)	(-3.50)	(-1.47)	(-0.88)	(-3.33)	(-1.43)	(-0.28)	(-3.77)	(-0.76)	(-0.89)	(-0.81)	(-3.42)
	Capital Reserve	0.025***	-0.001*	0.023***	0.024***	-0.002***	0.022***	0.033***	-0.002***	0.031***	0.033***	0.024***	-0.002***
		(4.60)	(-2.42)	(4.32)	(4.47)	(-3.01)	(4.10)	(6.11)	(-4.23)	(5.68)	(5.99)	(4.43)	(-3.03)
	Current Assets	-0.061***	-0.005***	-0.068***	-0.060***	-0.005***	-0.066***	-0.071***	-0.004***	-0.075***	-0.075***	-0.060***	-0.005***
		(-7.94)	(-5.72)	(-8.78)	(-7.75)	(-5.48)	(-8.48)	(-9.14)	(-4.72)	(-9.68)	(-9.75)	(-7.85)	(-5.47)
Year Fixed Effect		Y	Y	Y	Y	Y	Y	Y	Y	Y	Y	Y	Y
Firm Fixed Effect		Y	Y	Y	Y	Y	Y	Y	Y	Y	Y	Y	Y
Observation		2061	2061	2061	2061	2061	2061	2030	2030	2030	2030	2061	2061
adj. R^2		0.336	0.204	0.347	0.338	0.188	0.347	0.353	0.190	0.359	0.364	0.335	0.188

#significance at 0.10, *significance at 0.05, **significance at 0.01, ***significance at 0.001. The *t* value is enclosed in parentheses. *Controls* include control variables, year fixed effect, and firm fixed effect.

6. Using professional proofreading to improve comprehensive academic writing style and the quality of communication

Response: Following the reviewers' advice, we carefully reviewed the manuscript, and the full text was re-combed, such as:

"Panel A of Table 2 shows that the coefficient of *Gender* is positive and significant at 0.01, which is consistent with the conclusion that the increased proportion of female executives promotes *CSR* performance in literature. As the present proportion of female executives in listed companies is relatively small, they pay more attention to their social identity and personal accomplishment by *CSR* implementation. The mediator impact of *CPDI* has a significant positive correlation with female *Gender* characteristics (β=0.017, p<0.05, Model 2). It was indicated that female executives prefer stable structures, or they are not good at changing corporate governance. And it has a negative effect on *CSR* and is significant at 0.01 (Model 3). This support the Intrinsic Nature of CEOs towards *CSR* for Hypothesis H1. "

For modifications to the full text, see the stamped attachment.

Also, we changed the word type to Latex type as requested by the journal. We used Latex to organize all the contents of the paper, as shown in the appendix of the paper.

7. Minor errors need to be solved:

In Hypothesis 1, there is a mistake after H1 "," (lines 102-103).

In the conceptual model, there is an overlapping of the arrows (Figure 1)

Table 1 can start at page 6, instead of the ending of the previous page.

Response: The Hypothesis and Figures have been modified.

In the paper, the article segmentation, table layout, and graphic location have been following the requirements of the journal. we use Latex software to adjust the formats to match the journal requirements.
