# Supplementary material for: The decentralization effects of entrepreneurial characteristics on corporate social responsibility
Source: PLoS One. 2022 Nov 28;17(11):e0278030. doi: 10.1371/journal.pone.0278030 (PMC9704557; doi:10.1371/journal.pone.0278030)
Supplement: Attachment — Submitted filename: Response to Reviewers.docx [file pone.0278030.s002.docx]

**Response to Reviewers**

Dear Editors and Reviewers:

Thank you for your letter and the reviewer's comments concerning our manuscript entitled “The decentralization effects of entrepreneurial characteristics on corporate social responsibility” (PONE-D-22-16972). Those comments are all valuable and very helpful for revising and improving our paper, as well as the essential guiding significance to our research. We have studied the words carefully and made a correction that we hope meets with approval. Revised portions are marked in red on the paper. The significant disciplines in the form and the responses to the reviewer's comments are as follows:

**1. The literature review part should incorporate recent research on the topic from the year 2021-2022, you may benefit from the following studies.**

https://doi.org/10.3390/su131910662

https://doi.org/10.3389/fpsyg.2022.841163

https://doi.org/10.3389/fpsyg.2022.897444

**Response:** Thanks very much for the references provided by the reviewers. We downloaded the above three contacts and read them carefully. These papers have studied Corporate Social Responsibility Performance (CSRP) from different aspects, mainly as follows:

(i) CEO characteristics positively moderate the relationship between corporate governance (CG) and social and environmental accountability (SEA).

(ii) Corporate Financial Performance (CFP) has a significant positive impact on CSRP, negatively moderated by CEO turnover and CEO duality.

(iii) Tournament incentives motivate CEOs to be more socially responsible for CSRP, which will be positively affected through sub-national institutional contingencies.

The above contents are of reference value to the contents we have studied. Therefore, the paper has been revised, and three articles are attached to the references. The specific modifications are as follows:

“The research results of Khan et al. [28] provide reliable evidence that tournament incentives motivate CEOs to be more socially responsible. ”

“The study by Zahid et al. [31] also found that CEO turnover and duality negatively moderate the relationship between corporate financial performance and CSR performance. Therefore, we must discuss the effectiveness of incentive features on CSR performance.”

“Khan et al. [36] found that the environmental performance of Chinese enterprises is affected by board size, independence, gender diversity, and CEO duality, which positively moderate the relationship between corporate governance and CSR. ”

2. **In the conclusion part the implications looks very generic, make it specific to context based on the findings of your studies.**

**Response:** We have revised the conclusion as follows; see attachment of “Revised Manuscript with Track Changes”.

**Conclusions and Policy Implementations**

In this study, we used the personal data of CEOs and the financial data of listed companies to research the influence of Entrepreneurial Characteristics (EC) on Corporate Social Responsibility (CSR) with the moderating role of the Corporate Power Distribution Index (CPDI) based on the Upper Echelons Theory. In terms of emotion, morality, and value, we found that the Academy, Salary, and female Gender of EC have significant positive effects on CSR performance. Under the moderators of Age, Shares, and Academy characteristics, CPDI can significantly mediate the relationship between EC and CSR. Moreover, Politics background significantly mediates the impact of a Degree on CSR. The result provides that corporate strategy should optimize the power distribution of internal governance structure from entrepreneurs' self-interest to solve the adverse CSR events. As entrepreneurs often utilize their capacity and interests to carry out CSR activities, both the Incentive Feature and the Experience Characteristics can stimulate their responsibility to promote CSR performance. So there are two ways to improve CSR performance:

On the one hand, it doesn't mean enterprises give up pursuing benefits to fulfill social responsibility activities. In contrast, they ought to optimize the corporate governance structure of the board of directors, supervisors, and managers to improve power distribution to avoid adverse CSR events even better for benefits. These checks and balances are affected by entrepreneurial characteristics, which adjust the corporate governance structure from different aspects of improving the educational learning degree of senior executives, introducing directors with academic or political backgrounds, and adequately allocating reasonable gender ratios. In addition, we should implement the incentive system with a management shareholding plan to reduce adverse CSR events; only when entrepreneurs know that the value is greater than the loss caused by CSR activities will it improve CSR performance correctly and efficiently.

On the other hand, the governance of adverse CSR events should look into its root cause and symptoms of the problems for the regulatory authorities. In a short time, what plays an influential role is to give entrepreneurs material incentives, such as salary and equity, to encourage them to improve their CSR performance more actively and reward them for doing well. At the same time, regulators should note that CSR performance reflects more corporate ethics and entrepreneurship, which requires an arduous long-term project at the moral level. Therefore, the government ought to design a set of incentive and restraint mechanisms for entrepreneurs and establish a unique entrepreneurial credit evaluation system by combining morality, interests, and law to promote the enterprises to integrate CSR performance into corporate performance.

**※** Please review your reference list to ensure that it is complete and correct. If you have cited papers that have been retracted, please include the rationale for doing so in the manuscript text, or remove these references and replace them with relevant current references. Any changes to the reference list should be mentioned in the rebuttal letter that accompanies your revised manuscript. If you need to cite a retracted article, indicate the article’s retracted status in the References list and also include a citation and full reference for the retraction notice.

**Response:** Thanks very much for your reminder. We proofread part of the paper's literature and references one by one. Then we replaced the problematic literature and added some valuable literature as follows.

1.Simon HA. Rational decision making in business organizations. The American Economic Review. 1979; 69: 493-513. http://www.jstor.org/stable/1808698

9.Kabir R, Thai HM. Does corporate governance shape the relationship between corporate social responsibility and financial performance? Pacific Accounting Review. 2017; 29: 227-258. https://doi.org/10.1108/PAR-10-2016-0091

10.Jackson SE, May KE, Whitney K. Understanding the dynamics of diversity in decision-making teams. Team Effectiveness and Decision Making in Organizations. 1995; 204: 203-261. https://www.researchgate.net/publication/275714101

12.Huang H, Zhao Z. The influence of political connection on corporate social responsibility: Evidence from listed private companies in China. International Journal of Corporate Social Responsibility. 2016; 1: 1-19. https://doi.org/10.1186/s40991-016-0007-3

14.Martínez JB, Fernández ML, Fernández PMR. Corporate social responsibility: Evolution through institutional and stakeholder perspectives. European journal of management and business economics. 2016; 25: 8-14. https://doi.org/10.1016/j.redee.2015.11.002

22.Beji R, Yousfi O, Loukil N, Omri A. Board diversity and corporate social responsibility: Empirical evidence from France. Journal of Business Ethics, 2021; 173: 133-155. https://doi.org/10.1007/s10551-020-04522-4

26.Martínez JB, Fernández ML, Moreno CM, Fernández PMR. Corporate social responsibility in the process of attracting college graduates. Corporate Social Responsibility and Environmental Management, 2015; 22: 408-423. https://doi.org/10.1002/csr.1355

28.Khan M K, Ali S, Zahid R M A, et al. Does whipping tournament incentives spur CSR performance? An empirical evidence from Chinese Sub-national Institutional Contingencies. Frontiers in psychology. 2022; 13: 841163. https://doi.org/10.3389/fpsyg.2022.841163

30.Liu J, Zhu Y. Executive shareholding, internal control and inefficient input of corporate social responsibility. Journal of Shandong University of Finance and Economics. 2018; 30: 55-65. https://doi.org/10.3969/j.issn.1008-2670.2018.06.007

31.Zahid RMA, Khurshid M, Khan W. Do chief executives matter in corporate financial and social responsibility performance nexus? A dynamic model analysis of Chinese firms. Frontiers in Psychology, 2022; 13. https://doi.org/10.3389/fpsyg.2022.897444

33.Ahmed AS, Duellman S. Managerial overconfidence and accounting conservatism. Journal of Accounting Research. 2013; 51: 1-30. https://doi.org/10.1111/j.1475-679X.2012.00467.x

37.Khan MK, Zahid RMA, Saleem A, Sági J. Board composition and social & environmental accountability: A dynamic model analysis of Chinese firms. Sustainability, 2021; 13: 10662. https://doi.org/10.3390/su131910662

**※** While revising your submission, please upload your figure files to the Preflight Analysis and Conversion Engine (PACE) digital diagnostic tool, https://pacev2.apexcovantage.com/. PACE helps ensure that figures meet PLOS requirements.

**Response:** The four pictures in the article have been uploaded to PACE and passed the inspection. And they are packaged into the *fig.tif* file.

Fig 1.

|  |  |  |
| --- | --- | --- |
| (a) *Shares* | (b) *Age* | (c) *Academy* |

Fig 2.
